# Adipose-Derived Extract Suppresses IL-1β-Induced Inflammatory Signaling Pathways in Human Chondrocytes and Ameliorates the Cartilage Destruction of Experimental Osteoarthritis in Rats

**DOI:** 10.3390/ijms22189781

**Published:** 2021-09-10

**Authors:** Hideki Ohashi, Keiichiro Nishida, Aki Yoshida, Yoshihisa Nasu, Ryuichi Nakahara, Yoshinori Matsumoto, Ayumu Takeshita, Daisuke Kaneda, Masanori Saeki, Toshifumi Ozaki

**Affiliations:** 1 Department of Orthopaedic Surgery, Okayama University Graduate School of Medicine, Dentistry and Pharmaceutical Sciences, Okayama 700-8558, Japan; hideki.kangeki0084@gmail.com (H.O.); akysda@gmail.com (A.Y.); ayumude2@hotmail.com (A.T.); dai_kaneda1020@yahoo.co.jp (D.K.); tozaki@md.okayama-u.ac.jp (T.O.); 2 Department of Orthopaedic Surgery, Okayama University Hospital, Okayama 700-8558, Japan; nasu_y@flute.ocn.ne.jp (Y.N.); pikumin55@gmail.com (R.N.); 3 Department of Nephrology, Rheumatology, Endocrinology and Metabolism, Okayama University Graduate School of Medicine, Dentistry and Pharmaceutical Sciences, Okayama 700-8558, Japan; yossy.m0629@nifty.com; 4 View Clinic Momonosato, Okayama 706-0224, Japan; saeki@momonosato.or.jp

**Keywords:** adipose tissue, cartilage, chondrocyte, osteoarthritis, IL-1 receptor type 2

## Abstract

We investigated the effects of adipose-derived extract (AE) on cultured chondrocytes and in vivo cartilage destruction. AE was prepared from human adipose tissues using a nonenzymatic approach. Cultured human chondrocytes were stimulated with interleukin-1 beta (IL-1β) with or without different concentrations of AE. The effects of co-treatment with AE on intracellular signaling pathways and their downstream gene and protein expressions were examined using real-time PCR, Western blotting, and immunofluorescence staining. Rat AE prepared from inguinal adipose tissues was intra-articularly delivered to the knee joints of rats with experimental osteoarthritis (OA), and the effect of AE on cartilage destruction was evaluated histologically. In vitro, co-treatment with IL-1β combined with AE reduced activation of the p38 and ERK mitogen-activated protein kinase (MAPK) pathway and nuclear translocation of the p65 subunit of nuclear factor-kappa B (NF-κB), and subsequently downregulated the expressions of matrix metalloproteinase (MMP)-1, MMP-3, MMP-13, a disintegrin and metalloproteinase with thrombospondin motifs (ADAMTS)-4, IL-6, and IL-8, whereas it markedly upregulated the expression of IL-1 receptor type 2 (IL-1R2) in chondrocytes. Intra-articular injection of homologous AE significantly ameliorated cartilage destruction six weeks postoperatively in the rat OA model. These results suggested that AE may exert a chondroprotective effect, at least in part, through modulation of the IL-1β-induced inflammatory signaling pathway by upregulation of IL-1R2 expression.

## 1. Introduction

Osteoarthritis (OA) is the most common form of chronic arthritis, is characterized by the destruction of articular cartilage, osteophyte formation, subchondral bone sclerosis, and secondary synovitis, and is expected to increase with the aging of the population. The Osteoarthritis Research Society International (OARSI) has recently endorsed a new definition, as follows: “Osteoarthritis manifests first as a molecular derangement (abnormal joint tissue metabolism) followed by anatomic, and/or physiologic derangements (characterized by cartilage degradation, bone remodeling, osteophyte formation, joint inflammation, and loss of normal joint function), that can culminate in illness” [1]. Currently, there are few effective and well-tolerated symptomatic treatments for OA other than joint replacement surgery, and there are no approved drugs that can alter the natural course of OA and provide long-term benefits [2]. Thus, the development of disease-modifying osteoarthritis drugs (DMOADs) are crucial challenges for better management of OA [3].

It is widely accepted that interleukin-1 beta (IL-1β) is one of the key cytokines that play critical roles in the progression of OA [4]. Binding of IL-1β to IL-1R1, a membrane receptor, activates transcription factors, such as MAPK and NF-*κ*B, resulting in a rapid induction of inflammatory mediators. Activation of these factors results in the expression of many genes, including *MMP* and *ADAMTS* metalloproteinases and inflammatory cytokines such as *IL-6* or *IL-8* [5]. Recently, an exploratory analysis of data from people who participated in the randomized controlled trial CANTOS using canakinumab, an antibody that inhibits IL-1β, showed that canakinumab reduced the percentage of patients who required total hip or knee replacement (THR/TKR) compared to the placebo group, supporting IL-1β inhibition in the treatment of OA [6]. 

Various attempts at tissue engineering were made in the field of regenerative medicine, and one of the highly anticipated therapies is transplantation medicine using adipose tissue [7,8]. Since it was reported that human adipose tissue contains adipose-derived stromal cells (ASCs) [9], adipose tissue, which can be harvested safely and in large quantities through liposuction, was considered to be an essential source of stem cells. The chondroprotective effects of ASCs were demonstrated in vitro [8,10,11,12,13] and in vivo [14,15], and clinical trials have shown the safety and efficacy of intra-articular injections of ASCs [7]. Mesenchymal stem cells (MSCs), including ASCs, are thought to act not only through direct differentiation into chondrocytes [16,17] but also through a paracrine mechanism that involves the secretion of bioactive factors that promote tissue regeneration processes [18,19,20,21]. However, the application of such a function to the treatment of OA necessitates overcoming some potential problems, including time and cost, residual toxicity of the collagenase, contamination, canceration, and restrictions associated with cell expansion and extensive manipulation [22,23]. In the last few years, micro-fragmented adipose tissue (MFAT) [24,25] and concentrated adipose tissue (CAT) [26,27,28] obtained without cell manipulation or enzymatic treatment have received much attention. CAT is often used for wound healing and tissue reconstruction purposes, mainly in the field of plastic surgery [29,30,31], whereas there are few reports of its use for OA treatment [32]. 

In the current study, we generated adipose-derived extract (AE) from CAT and examined the effects of AE on human chondrocytes stimulated with IL-1β in vitro. We also examined the effect of homologous AE on cartilage tissue in vivo by intra-articular injection into the knee joint of experimental OA in a rat model.

## 2. Results

### 2.1. The Effects of AE on IL-1β-Induced mRNA Expression 

**Figure 1:**
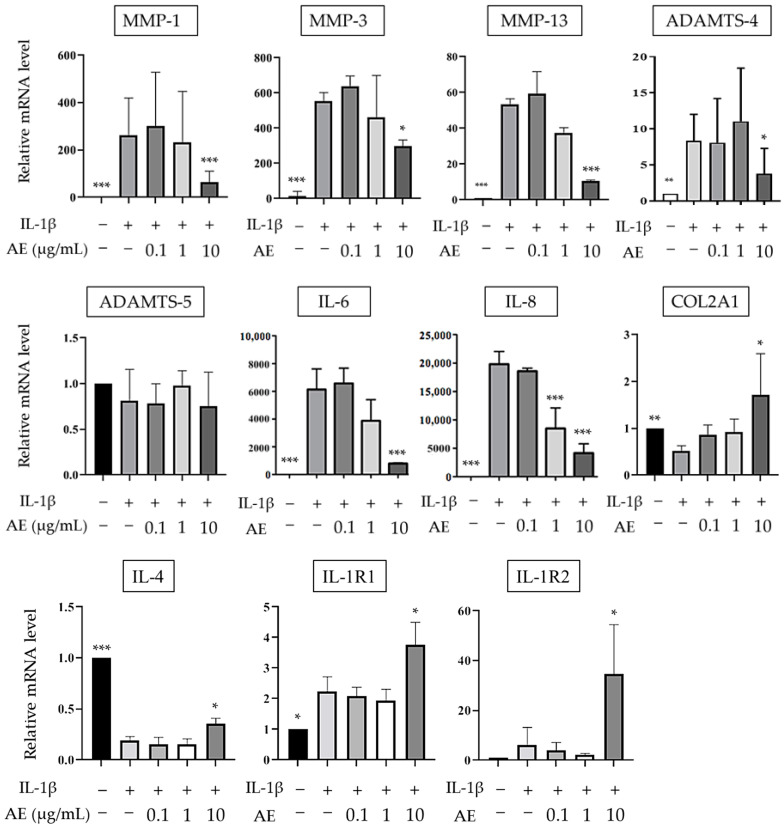
The effect of AE on IL-1β-stimulated mRNA expressions of anabolic and catabolic factors by NHCs. The results are expressed as the relative expression levels (mean ± SD) to control samples without IL-1β stimulation or AE treatment. Statistical significance: Tukey’s multiple comparisons test * *p* < 0.05, ** *p* < 0.01, *** *p* < 0.001 compared to IL-1 β stimulation without AE.

Real-time PCR (RT-PCR) was used to quantify the effects of AE on the mRNA expression of normal human articular chondrocytes (NHCs) stimulated with IL-1β. In NHCs, the expressions of *matrix metalloproteinase (MMP)-1*, *MMP-3*, *MMP-13*, *A Disintegrin and Metalloproteinase with Thrombospondin motifs (ADAMTS)-4*, *IL-6*, and *IL-8* were all significantly increased in the IL-1β alone group compared to the un-stimulated control cells. These gene expressions significantly decreased in cells treated with AE in a dose-dependent manner, although *ADAMTS-5* did not significantly change after AE treatment (Figure 1). There was no significant difference in mRNA expression between the group of chondrocytes stimulated with AE alone (without IL-1β) and the group of unstimulated control cells. In contrast, the expression of *COL2A1* and *IL-4* were significantly downregulated by IL-1β stimulation, and upregulated when cells were treated with IL-1β/AE (10 µg/mL). IL-1β stimulation increased the expression of both type 1 and type 2 *IL-1 receptor (IL-1R)*, and the treatment with AE resulted in a 3.8-fold and 35-fold increase in IL-1R1 and IL-1R2 expression, respectively (Figure 1).

### 2.2. The Effects of AE on the IL-1β-Induced Protein Expressions Involved in the Mitogen-Activated Protein Kinases (MAPK) Pathway

**Figure 2:**
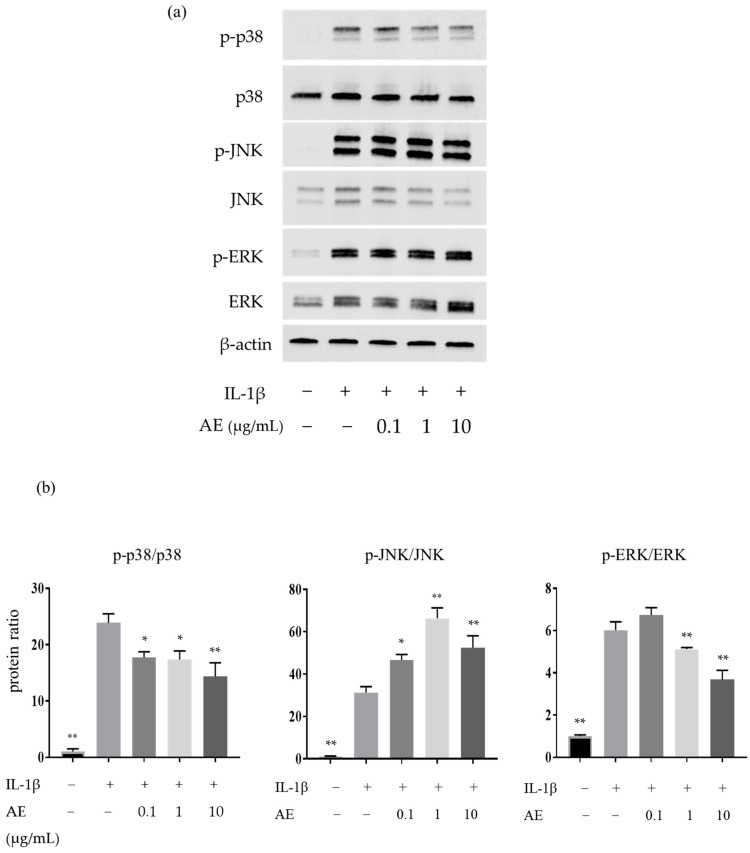
Western blot analysis of proteins involved in the MAPK pathway in NHCs. (**a**) Representative Western blots of total and phosphorylated MAPK family proteins. (**b**) Quantification of phosphorylated p38, JNK, and ERK. Statistical significance: Tukey’s multiple comparisons test * *p* < 0.05, ** *p* < 0.01 compared to IL-1 β stimulation without AE.

We investigated the effect of AE on the MAPK pathway by Western blotting. IL-1β significantly increased the protein expression of p-p38, p-JNK, and p-ERK, and AE reduced the IL-1β-induced protein expression of p-p38 at 0.1 µg/mL or higher and p-ERK at 1 µg/mL and 10 µg/mL, but increased p-JNK induced by IL-1β at 0.1 µg/mL or higher (Figure 2).

### 2.3. The Effect of AE on IL-1β-Induced Activation of the Transcription Factor Nuclear Factor-kappaB

**Figure 3:**
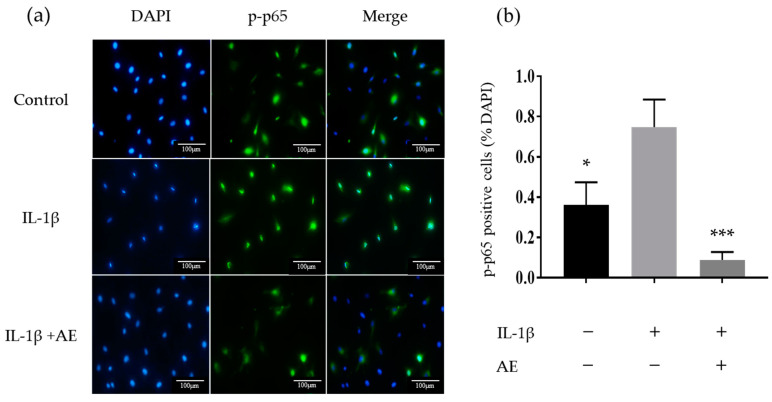
Immunocytochemistry of p-p65 expression. (**a**) Representative image showing the localization of p-p65 in chondrocytes. Original magnification ×200. (**b**) Percentages of p-p65 positive cells relative to total cell numbers. Data are shown as the mean ± SD of five fields. Statistical significance: Tukey’s multiple comparisons test * *p* < 0.05, *** *p* < 0.001 relative to the result of IL-1 βstimulation without AE.

We investigated the effect of AE on the activation of the transcription factor, nuclear factor-kappaB (NF-κB) using immunofluorescence. IL-1β stimulation activated NF-κB and induced nuclear translocation of the p-p65 subunit, while co-administration of IL-1β and AE suppressed the p-p65 nuclear translocation (Figure 3a). Figure 3b shows the results of immunofluorescence staining, expressed as the percentage of p-p65 positive cell nuclei counted in five fields. The percentage of p-p65-positive cells was significantly increased by IL-1β stimulation, and AE treatment significantly reduced the number of p-p65 positive cells. 

### 2.4. Effect of AE on the Histological Evaluation of Cartilage of Experimental OA Model Rats

**Figure 4:**
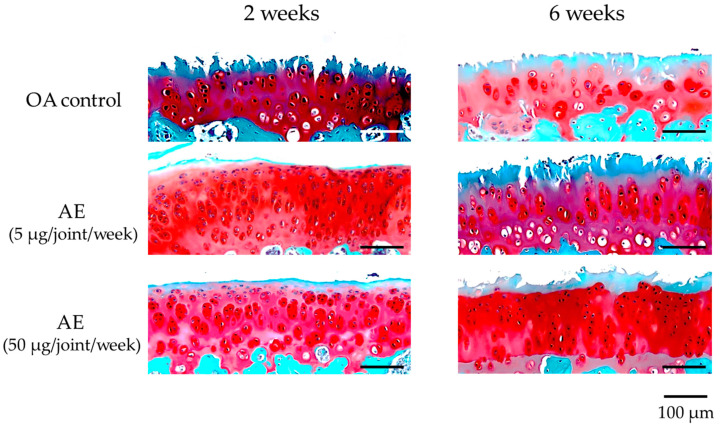
Histologic appearances of medial femoral condyle cartilage (safranin O staining) of the right knee joint of experimental OA model rats. Samples were obtained at 2 and 6 weeks after surgery from the joints without treatment (OA control, *n* = 18), and with lower dose AE treatment (5 µg/joint/week, *n* = 20) and higher dose AE treatment (50 µg/joint/week, *n* = 19). Progression of cartilage destruction was milder in the treatment group than in the control group at 6 weeks after surgery.

On safranin O-stained sections of the femoral condyle, OA control sections showed mild–severe progression of cartilage destruction with loss of the superficial layer, surface fibrillation, and decreased numbers of chondrocytes 2 weeks after surgery. By 6 weeks after surgery, the cartilage lesions extended to the middle layer, with decreased staining of safranin O and decreased numbers of chondrocytes. Sections from the group treated with AE at 2 weeks showed mild cartilage deterioration with decreased safranin O staining at the superficial layer, increased numbers of chondrocytes and cluster formation. At 6 weeks after surgery, sections from the group treated with AE (5 µg/joint/week) showed loss of the superficial layer, surface fibrillation, and decreased numbers of chondrocytes, whereas the sections from the group treated with AE (50 µg/joint/week) showed less severe cartilage degeneration with loss of safranin O staining at the superficial layer and decreased numbers of chondrocytes (Figure 4). There was no significant difference in the modified Mankin scores between the OA control group (4.33 ± 1.32) and the AE treatment groups (AE 5 µg: 3.20 ± 2.30, AE 50 µg: 2.78 ± 1.09) at 2 weeks after surgery. The modified Mankin scores had increased significantly at 6 weeks (6.89 ± 2.02) compared with those at 2 weeks in the OA-control group. The histologic scores of the AE treatment group sections were significantly lower than those of OA control group sections at 6 weeks (AE 5 µg: 4.20 ± 2.30, AE 50 µg: 3.10 ± 1.97), suggesting that intra-articular injection of AE inhibited the progression of cartilage destruction 6 weeks after surgery (Table 1). A dose-dependent effect of AE on the inhibition of cartilage destruction was not found in the present sets of concentrations of AE. Sections of femoral condyles from the left knee after sham operation showed minimal changes in the modified Mankin scores through the subsequent time course following arthrotomy.

## 3. Discussion

Currently, the potential mechanisms of stem cells in articular cartilage regeneration can be divided into two main categories: the “differentiation theory” and “paracrine theory” [33]. Differentiation theory is an approach to articular cartilage regeneration therapy in which stem cells directly differentiate into chondrocytes. However, it is still difficult to regenerate hyaline cartilage in a stable manner. Studies based on differentiation theory often report that tissues after cell transplantation in vivo show signs of type I collagen-rich fibrous cartilage and, in vitro, there are scattered reports that when MSCs are induced to differentiate against chondrocytes, they differentiate into hypertrophic chondrocytes expressing type X collagen [34]. On the other hand, for the paracrine theory, MSCs were shown to secrete a number of bioactive factors, extracellular vesicles, and extracellular matrix for local miniaturization and remodeling. De Windt et al. [35] reported that histological analysis of cartilage tissue regenerated by allogeneic MSCs transplantation showed hyaluronic acid-like regeneration with high concentrations of proteoglycans and type II collagen. Interestingly, when the histological samples were examined, no allogeneic MSC DNA was detected in the repaired tissue, indicating that the transplanted MSCs provided the initial stimulus but were subsequently removed from the tissue. These studies suggest that the function of stem cells in tissue repair and regeneration might be largely mediated by the paracrine mechanism.

We investigated the effect of intra-articular injection of AE prepared from allogeneic adipose tissues on the progression of cartilage degeneration in a rat experimental OA model. The results of the current study confirmed that the cartilage damage progressed over time in the control group, while it was suppressed at 6 weeks after surgery in the AE group, suggesting that homologous AE exerts a chondroprotective effect in a rat OA model. As AE is considered as non-cellular and, therefore, non-immunogenic, and was shown to be cryopreservable [31], the chondroprotective effect of allogeneic AE on the rat OA model shown in this study may enable the use of AE with homologous or heterologous proteins in the future.

In the current study, we demonstrated that AE suppressed IL-1β-induced activation of intracellular signaling pathways and downstream effects and upregulated the expression of *COL2A1*. Our results are consistent with the report of Tofino-Vian et al. [12], who found that ASC-conditioned medium (ASC-CM) counteracted the effects of IL-1β in chondrocytes, and with a report by Platas et al. [13], who found that the inhibitory effect of ASC-CM on the expression of catabolic and inflammatory molecules in chondrocytes stimulated by IL-1β was associated with reduced activation of NF-*κ*B. 

For ADAMTS-5, Koshy et al. [36] reported ADAMTS-4 mRNA is induced by catabolic cytokines, but ADAMTS-5 mRNA is not regulated by cytokines and is constitutively expressed in human chondrocytes. Naito et al. [37] reported that ADAMTS-5 is constitutively expressed in human normal and OA cartilage, and ADAMTS-4 protein is overexpressed in OA cartilage with a direct correlation to the degree of cartilage destruction. These data suggest that ADAMTS-4, but not ADAMTS-5, may play a major role in aggrecan degradation in human OA [37,38]. Our data confirm these differences in the response of ADAMTS-4 and ADAMTS-5 to cytokine stimulation in human chondrocytes, as stimulation of human chondrocytes with IL-1β or AE did not significantly alter ADAMTS-5 expression in this study.

The MAPK pathways are responsible for the conversion of a large number of extracellular stimuli into specific cellular responses that range from positive and negative roles in cell proliferation, differentiation, and apoptosis to the regulation of inflammatory and stress responses. All of the MAPK pathways are organized into cascades, and the JNK pathway is mainly activated by cellular stress and cytokines through several upstream kinases [39,40]. Kamata et al. [41] reported that the NF-κB-JNK crosstalk, in which JNK activation is prolonged when NF-κB is suppressed, is mediated by an alternative pathway of JNK activation by reactive oxygen species. Greene et al. [42] stimulated monolayer cultures of primary human articular chondrocytes with IL-1β and reported that the response pattern of each signaling protein was different, with p38 reaching peak phosphorylation in 10–15 min and ERK and JNK in 10–30 min. In the present study, the expression of p-JNK, which was upregulated by IL-1β, was further upregulated by the addition of AE. A possible explanation might be that the suppression of NF-κB by AE could lead to the sustained activation of JNK and alter its response pattern.

Furthermore, our in vitro study included important results revealing that the expression of *IL-4* was severely inhibited by the addition of IL-1β, but significantly upregulated by the addition of AE. A number of studies have reported that *IL-4* has an inhibitory effect on the degradation of proteoglycans in articular cartilage by inhibiting the secretion of MMPs and reducing the variability in proteoglycan production seen during the course of OA, suggesting that IL-4 is associated with a strong chondroprotective effect [4]. For *IL-1R*, *IL-1R2* expression was more significantly upregulated compared to *IL-1R1* in the group co-stimulated with IL-1β and AE (10 µg/mL). IL-1R2 is a receptor that binds to IL-1 ligands and was defined as a decoy receptor. IL-1R2 does not show the ability to transduce and activate intracellular signals when bound to IL-1β, representing one of the significant mechanisms of inhibition of IL-1 activity [43]. Additionally, IL-1R2 has membrane-bound and soluble forms (sIL-1R2), and sIL-1R2 is considered as a very efficient IL-1 inhibitor because sIL-1R2 is as effective as its membrane receptor in binding to IL-1β, while it lacks the ability to bind the antagonist IL-1Ra and has the unique characteristic of efficiently binding to pro-IL-1β, an inactive precursor of IL-1β [44]. Colotta et al. [45] reported that IL-4 antagonized the action of IL-1 on human neutrophils by inducing the expression and release of IL-1R2. Our results suggested that AE may upregulate IL-1R2 expression resulting in inhibition of IL-1β activity the through upregulation of IL-4.

A number of studies have demonstrated the availability of adipose tissue without cell expansion or enzymatic treatment [25,26,27,28,46,47,48,49,50,51,52]. MFAT is the tissue obtained from lipoaspirate using an isolation and washing device without the use of enzymes. MFAT retains the adipose niche, which includes the extracellular matrix (ECM) in addition to various cells, such as ASCs, endothelial cells, and pericytes [25,46,47,48,49,50,51,52]. Hudetz et al. [46] considered that in MFAT transplantation, ASCs and pericytes remain viable and effective within the conserved adipose niche, sensing the ambient environment of the knee joint and initiating the secretion of bioactive factors. Desando et al. [51] surmised that the collagen fiber network structure of MFAT evades enzymatic degradation, allowing for the long-term survival of the included cells and the gradual release of cytokines.

On the other hand, CAT is a concentrated adipose tissue obtained from lipoaspirate by centrifugation without cell expansion or enzymatic treatment [26,27,28]. Centrifugation is performed to separate blood cell components and oils from lipoaspirate, and it was shown that clusters of adipocytes and ASCs in tissues are well preserved when centrifuged below 3000× *g* [27]. This means that CAT retains the adipose niche containing various cells and ECM, and is expected to have the same effect as MFAT. However, since AE does not contain cells, the effect of ASCs on secreting large amounts of bioactive molecules in response to environmental sensing, which has attracted the attention of many scientists, cannot be expected. During the past two decades, adipose tissue has come to be considered an endocrine organ that not only functions as a fat and energy reservoir but also secretes paracrine factors that regulate a variety of physiological functions [53,54]. Carelli et al. [47] showed that mechanical activation abolishes the inflammatory properties of adipose tissue and promotes its expression of anti-inflammatory proteins. It might be reasonable to assume that mechanical treatment of adipose tissue led to a rapid increase in the initial secretion and storage of bioactive factors, which were extracted into AE by centrifugation, as reported by He et al. [31] Although the life span of these bioactive factors is expected to be short, it was suggested that they exert their effects in vivo by activating a cascade of reactions [19,55,56]. 

The current study had some limitations. First, because liposuction is difficult in rats, rat adipose tissues were not precisely the same as the lipoaspirates of humans. In this study, we treated adipose tissue obtained by mechanical shredding alone as equivalent to adipose tissue obtained without cell expansion or enzymatic treatment. Second, in this study, we considered AE to be susceptible to clearance from the joint space and consequently we performed weekly intra-articular injections and did not compare them to a single injection. Comparisons of the duration of chondroprotective effects of adipose tissue versus its extract is essential information for optimizing the clinical application of adipose-derived tissue in OA treatment. Lastly, our in vitro study was poorly analyzed at the protein level and did not lead to the identification of specific bioactive factors. Proteomic analysis of microvesicles and exosome fractions of ASC-CM has shown that they contain unique proteins, some of which were reported to be involved in the regulation of inflammatory processes and immune responses [12]. Nava et al. [52] suggested that the potent anti-inflammatory activity of MFAT is a highly complex phenomenon that depends on the combination of molecules and extracellular vesicles secreted by ASCs and endothelial cells. They showed that MFAT produces high levels of granulocyte colony stimulating factor (G-CSF), stem cell growth factor beta, and hepatocyte growth factor (HGF), and stated that G-CSF stimulates the production and activation of MSCs, induces increased expression of HGF, and improves tissue recruitment capacity and anti-inflammatory status. They also stated that G-CSF production in MFAT could be associated to the activation of endothelial cells probably due to the shearing force produced during MFAT regulation. The chondroprotective mechanisms of MFAT, CAT, and their secretions need to be elucidated by further studies.

In contrast, there are several advantages of AE for clinical application. AE can be prepared quickly and cleanly using only centrifugation, so it can be used safely at low cost during surgery. Furthermore, because it is not a cell-based therapy, it is not immunogenic, and there is a possibility that homologous or heterologous proteins can be used in the future. Lastly, it is easy to store and transport. This is the first study to examine the effects of AE, including the secretome of concentrated adipose tissue, prepared only by centrifugation, on cartilage tissue. We believe the results of the current study provide valuable information to optimize future OA treatments using adipose-derived tissues.

## 4. Materials and Methods

All the procedures, including animal studies, were conducted after receiving approval from Okayama University Institutional Review Board (1612-014; Approval date; 14 December 2016) and the Institutional Animal Care and Use Committee and approved by the President of Okayama University (OKU-2016245; Approval date; 22 June 2016).

### 4.1. AE Preparation 

**Figure 5:**
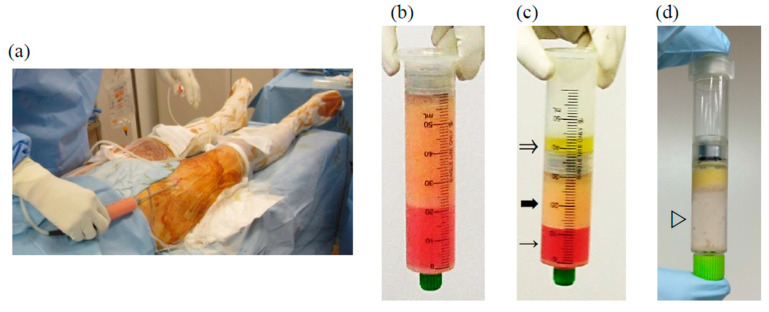
Preparation of adipose-derived extract (AE) (**a**) Liposuction from the donor’s thigh. (**b**) Lipoaspirate before centrifugation. (**c**) Adipose tissue after centrifugation separated into three fractions: oil (⇒), adipose tissue (➡), fluid (→) (**d**) AE (▷) obtained as the aqueous fraction.

Human adipose tissues were obtained from four female donors aged no less than 18 years-old, undergoing cosmetic liposuction from the thigh at the related facility after obtaining written informed consent (Figure 5). Liposuction from the thigh was not indicated to the patients with BMI less than 17.5 or greater than 35 kg/m^2^, previous thigh surgery, comorbidities, or regular medicine that prohibit the surgery or anesthesia. The mean age and body mass index (BMI) of donors were 31.7 (range: 24–37) years and 20.4 (range: 17.9–22.5) kg/m^2^, respectively. The lipoaspirates were poured into disposable sterilized 50 mL syringes with a filter piston (Medikan Corp., Seoul, Korea) and centrifuged at 1200× *g* for 3 min using a Lipokit^®^ device (Medikan) [27]. After centrifugation, samples were separated into three fractions: oil (onto the piston), adipose (middle: concentrated adipose tissue), and fluid (bottom: containing the blood cell component). The concentrated adipose tissue was collected and mixed with an equal volume of PBS. AE was obtained by centrifuging at 2600× *g* for 10 min (Model 2800, Kubota Co., Tokyo, Japan), then filtering the aqueous portion and removing cellular and tissue debris. The protein concentration of AE was measured using a Bradford Protein Assay Kit (TaKaRa Bio Inc., Shiga, Japan). All the procedures were performed under sterile conditions.

### 4.2. Chondrocyte Cultures

NHCs from knee cells obtained from a 15-year-old male, a 34-year-old-male, and a 38-year-old-male were purchased from Lonza (Walkersville, MD, USA). Cells were cultured at 37 °C in 5% CO_2_ in CGM™ Chondrocyte Growth Medium BulletKit™ (Lonza; CBM™ Chondrocyte Growth Basal Medium (Lonza) with CGM™ Chondrocyte Growth Medium SingleQuots™ Supplements and Growth Factors (Lonza)) containing supplements and several growth factors [R3 insulin-like growth factor (R3-IGF-1), basic fibroblast growth factor (bFGF), tranferrin, insulin, fetal bovine serum (FBS), and gentamicin/amphotericin-B]. The medium was changed every 3 days, when the cultures reached sub-confluence, the cells were subcultured at split ratios of 1:3 using Accutase (Innovative Cell Technologies, San Diego, CA, USA), and NHCs were used at passage 3 (P3). NHCs were seeded in 6-well plates at 2 × 10^5^ cells per well and cultured in 2 mL CGM. For all experiments, after 24 h and confirmation that the NHCs had adhered to the 6-well plates, all the medium was aspirated, and the medium was replaced by CBM containing 1% FBS.

### 4.3. Real-Time PCR

For RT-PCR, NHCs (P3) plated into 6-well plates at 2 × 10^5^ cells per well were treated with IL-1β (10 ng/mL, PeproTech, Inc., Rocky Hill, NJ, USA) with or without different doses of AE (0, 0.1, 1, or 10 µg/mL protein concentration) for 30 min. Total RNA was extracted from NHCs using QIAzol Lysis Reagent (Qiagen, Valencia, CA, USA) according to the manufacturer’s instructions. Reverse transcription was accomplished on 500 ng of total RNA using Primescript RT master mix (Takara Bio). RT-PCR was performed using an Agilent Mx3000P instrument (Agilent Technologies, Santa Clara, CA, USA) according to the manufacturer’s instructions. Reaction components were prepared to a final concentration as follows: 5.0 µL of TaqMan 2× Universal PCR Master mix, 0.50 µL of each primer, and 2.0 µL cDNA. The primers were as follows for TaqMan^®^ Gene Expression Assays (Applied Biosystems, Foster City, CA, USA): *COL2A1*; Hs00264051_m1, *MMP-1*; HS00899658_m1, *MMP-3*; Hs00899658_m1, *MMP-13*; Hs00233992_m1, *IL-4*; Hs00174122_m1, *IL-6*; Hs00174131_m1, *IL-8*; Hs00174103_m1, *IL-1R1*; Hs00991010_m1, *IL-1R2*; Hs0074759_m1, *ADAMTS-4*; Hs00192708_m1, and *ADAMTS-5*; Hs01095518_m1, and GAPDH; Hs02786624_g1 and the final expression levels were calculated by dividing the expression levels of each gene by the expression level of *GAPDH*. The primer of *IL-1R2* used in this study was designed to straddle Exons 6–7, enabling detection of both membrane and soluble types. Each value obtained for the control cells was set to one. 

### 4.4. Western Blotting

For the Western blot analysis, NHCs (P3) plated into 6-well plates at 2 × 10^5^ cells per well were treated with IL-1β (10 ng/mL) with or without different doses of AE (0, 0.1, 1, or 10 μg/mL protein concentration) for 30 min. Total protein was extracted, and the concentration was determined using a Bradford Protein Assay Kit (TaKaRa Bio Inc.). Total proteins were loaded and separated on a Mini-Protean^®^ Tris-glycine extended gel (Bio-Rad, Richmond, CA, USA) and transferred onto an Immun-Blot^®^ PVDF membrane (Bio-Rad). Blots were blocked in Odyssey blocking buffer (LI-COR, Lincoln, NE, USA) for 1 h at room temperature. Following washing, blots were incubated overnight at 4 °C with the following primary antibodies: 1:1000 anti-ERK1/2 antibody (Cell Signaling Technology, Danvers, MA, USA); 1:1000 ERK1/2 phospho (Thr202/Tyr204) antibody; 1:1000 p38 MAPK antibody; and 1:1000 p38MAPK phospho (Thr180/Tyr182) antibody. After washing three times in TBS containing 0.1% Tween-20 (TBS-T) for 5 min each, blots were incubated with IRDye^®^ 800CW anti-mouse or IRDye^®^ 680CW anti-rabbit secondary antibody (LI-COR) for 1 h at room temperature and then washed three times in TBS-T for 5 min each. Band intensity was analyzed using a LI-COR Odyssey infrared fluorescence scanning imaging system and quantified using Odyssey infrared imaging system application software version 2.1. The relative expression of each protein was calculated as the ratio between phosphorylated and total protein.

### 4.5. Immunocytochemistry

For immunocytochemistry, NHCs (P3) were plated into 8-well chamber slides at 4 × 10^3^ cells per well and cells were grown to subconfluence, then serum-starved in chondrocyte basal medium (Lonza) containing 1% FBS for 24 h. The cells were then treated with IL-1β (10 ng/mL) alone, or IL-1β (10 ng/mL) with AE (10 µg/mL) or PBS (as control) for 1 h. After treatment, cells were fixed with 1% paraformaldehyde in PBS for 10 min at 4 °C, blocked with 1% BSA in PBS for 20 min at room temperature and incubated with phospho-NF-κB p65 (Ser536) (7F1) mouse mAb (Cell Signaling Technology, Danvers, MA, USA) followed by incubation with Alexa Fluor 488-conjugated goat anti-rabbit IgG (Abcam, Cambridge, UK). Slides were mounted in Prolong Gold antifade reagent with DAPI (Thermo Fisher Scientific, Waltham, MA, USA) and fluorescence images were obtained using a phase contrast microscope (IX73; Olympus, Tokyo, Japan) equipped with a dual CCD digital camera (DP80; Olympus). We evaluated the percentage of p-p65 positive cell nuclei in five fields of each slide for each condition.

### 4.6. Preparation of the Experimental OA Model

For the experimental OA model, 8-week-old male Wistar rats (Crlj:WI) (mean weight of 291.4 ± 5.7 g) were purchased from CLEA Japan Inc. (Tokyo, Japan). The animals were housed in specific pathogen-free cages with a maximum of two animals per cage, the room temperature was 22–24 °C, and food and water were freely available. No medication or treatment was administered prior to this experiment. Animals were anesthetized by inhalation administration of isoflurane and intraperitoneal injection of pentobarbital sodium (50 mg/kg). Experimental OA was induced in the right knee joints of rats. We used the same animal model of OA which was used in our previous study [57], first reported by Hayami et al. [58]. Briefly, the anterior cruciate ligament and medial collateral ligaments (ACL and MCL, respectively), as well as the medial meniscus were transected using the medial parapatellar approach. The knee joints of this model showed a relatively rapid and severe destruction of cartilage. Arthrotomy only was performed on the left knee joints as a sham operation. In this model of rats, intra-articular injection was accurately done because of the size of the knee joint, and animals had enough volume of adipose tissue for experiments in the groin region without individual differences.

### 4.7. Preparation and Intra-Articular Injection of Rat AE

Adipose tissues were harvested from the left inguinal zone of rats at the same time as the knee surgery. All adipose tissue was removed from the membrane and mechanically shredded using a surgical knife. Rat AE was obtained from adipose tissue in the same manner as described for human AE. The protein concentration of the sample was measured and samples were stored at −80 °C until use. The rats in the treatment group received a weekly intra-articular injection of 5 (*n* = 20) or 50 µg (*n* = 20) (as a protein)/50 µL of AE in the right knee joint and the rats in the control group (*n* = 20) received an intra-articular injection of 50 µL PBS as a vehicle in the same manner. Animals were anesthetized using isoflurane, and the vehicle or AE was injected into the knee joints from the center of the patellar tendon. We confirmed that the tip of the needle reached the joint space by loss of resistance or aspiration of joint fluid. The animals were sacrificed at 2 weeks (*n* = 28, average weight 354.6 ± 16.9 g) and 6 weeks (*n* = 29, average weight 472.2 ± 20.2 g) after surgery, and both knee samples were isolated for histological examination. For euthanasia, the animals were placed in a special container and the concentration of carbon dioxide gas was gradually increased by 20% per minute to 100% within 5 min. Three rats died during treatment as a result of the anesthesia. In several rats, we observed a subcutaneous leachate effusion in the fat harvesting area of the left groin, regardless of treatment. There were no adverse events associated with the intra-articular injection of AE in our rat OA model.

### 4.8. Tissue Preparation and Histological Evaluation

Histological evaluation was performed on full-thickness sagittal sections of cartilage in the weight-bearing area of the medial femoral condyle. The knee joint samples were dissected, fixed in 4% PFA for 24 h, and defatted in alcohol. Then, the knee joint samples were decalcified in 0.3 M ethylenediaminetetraacetate (EDTA; pH 7.5) for 14 days and embedded in paraffin^48^. Sections were stained with 0.1% safranin O. Histopathological classification of the severity of each OA lesion was graded on a scale of 0–13, using the modified Mankin scoring system [59,60]. The modified Mankin score is a combined score assessing structure (0–6 points), cellular abnormalities (0–3 points), and matrix staining (0–4 points).

### 4.9. Statistical Analysis

All data are expressed as the mean ± standard deviation (SD). Differences among individual sample groups were statistically analyzed by the Tukey’s multiple comparisons test. All analyses were conducted using GraphPad Prism 7 (GraphPad Software, San Diego, CA, USA) with a *p*-value < 0.05 regarded as significant.

## Figures and Tables

**Table 1:** Evaluation of cartilage degeneration by modified Mankin scores.

Operation Group	2 Weeks		6 Weeks	
	Modified Mankin Score	No. of Joints	Modified Mankin Score	No. of Joints
Sham	0.12 ± 0.33	28	0.08 ± 0.28	29
OA control	4.33 ± 1.32	9	6.89 ± 2.02 *	9
AE (5 μg/joint/week)	3.20 ± 2.30	10	4.20 ± 2.30 ^†^	10
AE (50 μg/joint/week)	2.78 ± 1.09	9	3.10 ± 1.97 ^†^	10

Statistical significance: Tukey’s multiple comparisons test. * *p* < 0.001 vs. same group at 2 weeks, ^†^ *p* < 0.001 vs. control OA group at 6 weeks.

## Data Availability

The data that support the findings of this study are available from the corresponding author, Keiichiro Nishida, upon reasonable request.
